# Demographic predictors of wellbeing in Carers of people with psychosis: secondary analysis of trial data

**DOI:** 10.1186/s12888-020-02691-0

**Published:** 2020-06-02

**Authors:** Cassie M. Hazell, Mark Hayward, Fiona Lobban, Aparajita Pandey, Vanessa Pinfold, Helen E. Smith, Christina J. Jones

**Affiliations:** 1 School of Social Sciences, University of Westminster, London, W1W 6UW UK; 2 Research and Development Department, Sussex Education Centre, Brighton and Sussex Medical School, Nevill Avenue, Hove, BN3 7HZ UK; 3 University of Sussex, Brighton, UK; 4 Research and Development Department, Sussex Partnership NHS Foundation Trust, Sussex Education Centre, Nevill Avenue, Hove, BN3 7HZ UK; 5 Health & Medicine, Lancaster University, Furness Building, Lancaster, LA1 4YX UK; 6 The McPin Foundation, 32-36 Loman Street, London, SE1 0EH UK; 7 Family Medicine and Primary Care, Lee Kong Chian School of Medicine, Nanyang Technological University Singapore, Singapore, 308232 Singapore; 8 School of Psychology, University of Surrey, Guildford, Surrey, GU2 7XH UK

**Keywords:** Carer, Psychosis, Mental health, Wellbeing, Demographic, Predictors

## Abstract

**Background:**

Carers of people with psychosis are at a greater risk of physical and mental health problems compared to the general population. Yet, not all carers will experience a decline in health. This predicament has provided the rationale for research studies exploring what factors predict poor wellbeing in carers of people with psychosis. Our study builds on previous research by testing the predictive value of demographic variables on carer wellbeing within a single regression model.

**Methods:**

To achieve this aim, we conducted secondary analysis on two trial data sets that were merged and recoded for the purposes of this study. Results: Contrary to our hypotheses, only carer gender and age predicted carer wellbeing; with lower levels of carer wellbeing being associated with being female or younger (aged under 50). However, the final regression model explained only 11% of the total variance.

**Conclusions:**

Suggestions for future research are discussed in light of the limitations inherent in secondary analysis studies. Further research is needed where sample sizes are sufficient to explore the interactive and additive impact of other predictor variables.

## Background

People, often family members or friends, who support another person to maintain a good quality of life, without payment, are referred to as carers [1]. More than 53 million people in the UK are currently carers [2].; they are a vital part of our health service. The support provided by carers to people with psychosis has been valued at £1.24 billion each year [3]. The evidence consistently indicates that carers are at an increased risk of developing their own physical and mental health problems compared to the general population [4–7] – however, this decline in health is not inevitable.

Not all carers of people with psychosis will want or require support. Amongst those carers who require support, their needs will differ and researchers have therefore endeavoured to identify which carers are likely to have the greatest necessity for support, in order that services can target their resources appropriately. Cross-sectional study designs have previously been used to explore factors which are associated with carer wellbeing, largely focussing on cognitive variables. For example, low carer wellbeing is associated with increased isolation [8–11], ineffective coping strategies [9, 11–13], and high self-blame [14].

There is some research exploring how the wellbeing of carers varies in relation to demographic variables. The factor with the greatest empirical support is what researchers have labelled the ‘burden of caring’; that is, those carers who are providing more care generally have the poorest wellbeing [13, 15–20]. Other demographic variables significantly associated with a decline in carers’ physical and mental health include being an older carer [12, 21], single [15], of lower educational attainment [13, 22, 23], and being in employment [24]. There are other factors where there is no evidence of their relationship with carer wellbeing; neither the care recipient’s diagnosis [20], whether the care recipient lived with the carer [25], nor how the carer is related to the care recipient [22] were significantly related to the carers’ health.

These studies have increased our knowledge of factors associated with distress and wellbeing in carers supporting people with psychosis. However, it is unclear whether any of these factors have an additive predictive effect on carer wellbeing, or even if they explain unique proportions of variance. Our analysis aims to address this limitation by entering multiple predictors into one, statistically powered, regression model. Moreover, this analysis will only use demographic variables as predictors, as opposed to some of the cognitive variables explored previously (e.g. self-blame, coping strategies). The rationale for confining our analysis to demographic characteristics is that these variables, more so than cognitions, align with a heuristic approach. That is, identifying what demographic variables predict carer wellbeing can have greater utility in clinical practice as services may be able to quickly and easily identify carers most in need for support at entry into the service, and target resources accordingly. Whilst NICE (2014) [26] guidance (QS80) recommend that all carers are offered a carer focussed education and support programme, and that structured Family Intervention is offered to all service users in contact with their families, the most recent data available from the Early Intervention in Psychosis Audit [27] show that services are not able to meet this target with current resources. A more precise knowledge of the carers at highest risk may help services to target their limited resources in the most productive manner.

### Aims

The aim of our study was to explore whether demographic variables predict wellbeing in carers of persons with psychosis by conducting secondary analysis of available trial data. Based on past research, we hypothesised that lower levels of carer wellbeing will be predicted by:

- Increased carer age (age)
- Longer duration of caring (duration of caring)
- Carer’s unemployment (employment status)
- Carer’s single status (relationship status)
- Lower educational attainment (educational attainment)

We also entered the following variables into the regression model as exploratory predictors: Carer’s ethnicity (ethnicity); carer’s gender (gender); care recipient’s diagnosis (care recipient’s diagnosis); whether the carer is living with the care recipient (living situation); and, how the carer is related to the care recipient (relationship to care recipient).

## Method

### Design

Our study is a secondary analysis of the baseline data collected across two randomised controlled trials (RCTs) of different interventions for carers of persons with psychosis. Both of the trials were UK-based pilot studies. One of the trials piloted a writing intervention for older adult carers (C4C) [28], and the other trial piloted the Relatives’ Education And Coping Toolkit (REACT) [29]. The REACT trial only recruited carers whose care recipient was a patient with Early Intervention in Psychosis (EiP) services, whereas the C4C trial did not exclude participants based on the type of service care recipients were receiving. Both trials used similar recruitment strategies (i.e. via mental health services, other health services, third sector, and self-referral). Due to word limit constraints, we refer readers to the respective study protocols for further information on the trial designs.

### Participants

All of the participants identified as carers, defined as a person who “provides unpaid support to a partner, child, relative or friend who couldn’t manage to live independently or whose health or wellbeing would deteriorate without this help” [1]. All participants were providing care to someone with psychosis which was defined as persons with a diagnosis of: schizophrenia, schizoaffective disorder, bipolar, depression with psychotic features, delusional disorder, psychosis not otherwise specified, first episode psychosis, and schizotypal personality disorder. Pooling the data from the two trials generated data from 165 carers for analysis (62 from the C4C trial, and 103 from the REACT trial). Participants completed the baseline assessments as part of the respective trials with the support of a member of the research team.

### Carer wellbeing scale v2 (CWSv2)

The CWSv2 [30] is a measure of wellbeing specific to carers. The CWSv2 has two sub-scales: (1) the wellbeing scale (measuring levels of carer wellbeing), and (2) the support scale (measuring the extent to which carers feel supported by mental health services) – in line with our hypotheses, we have only used the wellbeing scale as our dependent variable. The measure was co-developed by the Royal College of Psychiatrists and Rethink Mental Illness for carers of people with dementia and mental health difficulties, with input from carers. This measure uses a 5-point Likert scale (0 = A lot, 1 = Quite a bit; 2 = Moderately; 3 = A little; 4 = Not at all); where a higher mean score of all 32 items indicates better wellbeing. The original validation of the CWSv2 reported strong internal consistency of the wellbeing scale used in the present study (*α* = .96) [30], and this was replicated in the present data set (*α* = .95). This reliability could not be improved by removal of any of the items.

### Merging the datasets

To determine whether there were any differences in carer wellbeing between the two studies, we we conducted an independent samples t-test with the study as the independent variable (REACT versus C4C), and the total score on the CWSv2 [30] as the dependent variable. We found there was no significant differences between the two studies in terms of carer wellbeing, representing a small between-group effect size (*t* (163) = − 1.38, *p* = .17; Cohen’s *d* = 0.22). We can therefore conclude that it is appropriate to merge the two datasets.

The demographics variables from both trials needed to be recoded to ensure consistency across the data sets. We included 10 carer predictors in this regression model: (1) Age; (2) duration of caring; (3) employment status; (4) relationship status; (5) educational attainment; (6) ethnicity; (7) gender; (8) care recipient’s diagnosis; (9) living situation; (10) relationship to care recipient. All predictors, except duration of caring, were collected as categorical data. All of the categorical variables were condensed into binary variables so there were sufficient numbers of participants within each category. Each of these binary variables was dummy coded. See Table 1 for details of how we operationalised the predictors across the datasets, and the dummy codes.

**Table 1:** Predictor variables and how they have been operationalised across the data sets

Predictor	Description
Age	Age here refers to the carer’s age at the time of the assessment. One study collected age as a categorical variable. Age was operationalised as either (a) Under the age of 50, or (b) Aged 50 or over (Dummy Codes: Under the age of 50 = 1; Aged 50 or over = 0).
Gender	This predictor refers to the carer’s gender. Gender was operationalised as either (a) Male, or (b) Female (Dummy Codes: Female = 1; Male = 0).
Ethnicity	Most of the carers identified as White British. All other ethnic groups were pooled together in a group we have labelled ‘ethnic minorities’. We therefore separated ethnicity as either (a) White British, or (b) Ethnic Minorities (Dummy Codes: Ethnic Minorities = 1; White British = 0).
Employment Status	We divided carers as either (a) Currently Employed, or (b) Not in Employment. The first of these includes carers who were engaged in any amount of paid or voluntary employment; and the later includes carers who were unemployed, claiming unemployment benefits, on prolonged sick leave, retired, or not in work because they are a full-time carer (Dummy Codes: Not in Employment = 1; Currently Employed = 0).
Educational Attainment	Carers’ level of educational attainment was grouped by whether the carer had achieved a University-level qualification or not. That is, either (a) Higher Education, or (b) No Higher Education, respectively (Dummy Codes: No Higher Education = 1; Higher Education = 0).
Relationship to CR	There were a wide variety of carer-care recipient relational dynamics across the two trials. To reduce the number of groups we clustered carers by whether they were a (a) Parent to the CR, or (b) Other Relationship to the care recipient (Dummy Codes: Other Relationship to CR = 1; Parent to the CR = 0).
Care recipient Diagnosis	The term ‘psychosis’ in these trials was used in its broadest sense. We grouped carers based on whether they were caring for someone with a medically-defined psychosis diagnosis i.e. schizophrenia or schizoaffective disorder; or all other psychosis-related diagnoses. That is, either (a) Schizophrenia Spectrum, or (b) Other Psychosis Diagnosis (Dummy Codes: Other Psychosis Diagnosis = 1; Schizophrenia Spectrum = 0).
Living Situation	We separated carers according to whether they were living with their care recipient at the time of the baseline assessment, or not. We conceptualised this as either (a) Living with CR, or (b) Living separately from care recipient (Dummy Codes: Living with CR = 1; Living Without CR = 0).
Relationship Status	We divided carers are either being (a) In a relationship, or being (b) Single. The first of these groups included any carers who were married, in a civil partnership, or cohabiting. Whereas, the second of these groups included carers who were single, separated or divorced (Dummy Codes: Single = 1; In a Relationship = 0).
Duration of Caring	This was the only continuous predictor included in our model. The duration that participants had been providing care was conceptualised as either the time since the care recipient psychosis onset, or an explicit report of how long theyhad been a carer for – both measured in months.

### Ethics

Both the C4C trial (reference: 16/NW/0757) and the REACT trial (reference: 15/NW/0732) received ethical approval from the North-West Lancaster NHS Research Ethics Committees. Participants in both the C4C and REACT trials provided written informed consent for their data to be used for research purposes. To protect the identities of our participants, we assigned and referred to each participant using a unique ID number.

### Analysis plan

Data were first inspected to check all the assumptions associated with parametric tests were met. If any issues with bias occurred, we planned to correct this using either a transformation or bootstrapping. To address the aims of this research study, we conducted a hierarchical multiple regression. The variables that we hypothesised would predict carer wellbeing (Age, Relationship Status, Employment Status, Educational Attainment, and Duration of Caring) were entered into the first block of the regression model using the forced entry method. The remaining exploratory variables were entered in a second block using a forward selection stepwise entry method. For the second block, only predictors that explained a significant proportion of unique variance (*p* < .05) were retained. To manage uneven or small group sizes, significance testing was followed up with between-group effect size calculations (Hedges *g*).

#### Power calculation

We conducted a post-hoc power calculation for a multiple regression with 10 predictors, 165 participants, and sufficient power to detect a medium effect size (*ρ*^*2*^ = .13) with an *α =* 0.05. This power calculation produces a power value of 0.93, exceeding 0.80, the criterion for “good” statistical power [31].

##### Missing data

All 165 participants provided complete data on the dependent variable. Missing data occurred where participants did not provide complete demographic information. Missing data was not replaced, and instead cases were excluded from the regression model on a case-by-case basis (i.e. at the individual predictor level).

## Results

### Testing parametric assumptions

Visual inspection of normality plots showed the data and residuals were both normally distributed. The kurtosis Z-scores did not significantly differ from normal (*Z*s ≤ 1.65, *p*s > .05). For two of the predictors, there was some slight negative skew (*Z*s ≤ 2.11, *p*s < .05). All other skew Z-scores did not significantly differ from normal (*Z*s ≤ 1.93, *p*s > .05). There was no evidence of influential cases (Cook’s Distance = 0.01) [32], and residuals appear to be unrelated (Durbin-Watson = 2.10) [33]. Finally, there was no evidence of multicollinearity (VIF ≤ 1.29) [34]. The results of these tests therefore suggested that the data set met the assumptions for parametric tests, negating the need for transformations or bootstrapping.

### Carer characteristics

Overall, participants had “moderate” concerns related to their wellbeing (*M* = 2.39). The carers in our sample tended to be over 50 years old, female, White British, not in employment, with no Higher Education qualifications. Their care recipient tended to have a schizophrenia spectrum diagnosis, be living with the carer, and the relationship between the carer and care recipient was that of a parent and child. The duration that participants had been caring for varied substantially; the standard deviation around the mean (*M* = 100.12 months) was 111.79 (Table 2).

**Table 2:** Descriptive statistics of CWSv2 by participant demographics. *Note*: CR = care recipient; * = confidence intervals do not cross zero; *M* = mean CWSv2 separated by group; *SD* = standard deviation around the mean of the CWSv2 score separated by group

	***n(%)***	***M***	***SD***	Hedge’s ***g (95% CI)***
*Age*	165			.42 (.09, .75)*
Under 50 years old	54 (32.73)	2.15	1.01	
Aged 50 or over	111 (67.27)	2.51	0.78	
*Gender*	165			.52 (.14, .91)*
Male	34 (20.61)	2.75	0.72	
Female	131 (79.39)	2.30	0.89	
*Ethnicity*	165			.20 (−.38, .79)
White British	153 (92.73)	2.40	0.87	
Ethnic Minorities	12 (7.27)	2.22	1.01	
*Employment Status*	164			.17 (−.14, .48)
Currently employed	79 (48.17)	2.31	0.89	
Not in employment	85 (51.83)	2.46	0.87	
*Educational Attainment*	165			.03 (−.27, .34)
Higher Education	70 (42.42)	2.41	0.81	
No Higher Education	95 (57.58)	2.38	0.93	
*Relationship to CR*	165			.32 (−.06, .70)
Parent to the CR	132 (80.00)	2.33	0.90	
Other relationship to CR	33 (20.00)	2.61	0.75	
*Care recipient Diagnosis*	141			.05 (−.34, .43)
Schizophrenia Spectrum	107 (75.89)	2.40	0.83	
Other Psychosis Diagnosis	34 (24.11)	2.44	0.99	
*Living Situation*	165			.03 (−.35, .28)
Living with CR	103 (62.42)	2.40	0.87	
Living without CR	62 (37.58)	2.37	0.89	
*Relationship Status*	144			.34 (−.004, .68)
In a relationship	90 (62.50)	2.52	0.74	
Single	54 (37.50)	2.24	0.97	

### Hierarchical multiple regression

Overall, Model 1 did not significantly differ from the mean model (*F* (5, 119) = 2.11, *p* = .07), but when additional predictors were added, the regression model was significantly improved (*F* (1, 118) = 5.57, *p* = .02), and the overall model became significant (*F* (6, 118) = 2.75, *p* = .01). The final model explained 11.30% of the total variance.

Several non-significant predictors were retained in Model 1 as these variables were forced into the model. Educational attainment, duration of caring, and relationship and employment status did not significantly predict carer wellbeing (all *p*s > .05). For Model 2, the remaining predictors were entered using a Stepwise method. All predictors except gender were excluded as they were non-significant (Care Recipient Diagnosis: *t* = −.09, *p* = .93; Living Situation: *t* = .11, *p* = .91; Relationship to Care Recipient: *t* = .68, *p* = .50; Ethnicity: *t* = −.36, *p* = .72).

In the final regression model, only age and gender significantly predicted carer wellbeing. That is, being female compared to male, and being aged under 50 compared to 50 or over, was predictive of worse carer wellbeing. See Table 3 for the full regression model. This finding is supported by the between-groups effects sizes presented in Table 2. The effect sizes for age and gender were both in the medium range with confidence intervals that did not cross zero, while all other effect sizes were small.

**Table 3:** Hierarchical regression model of carer demographics predicting CWSv2. *Note:* Model 1 = forced entry; Model 2 = stepwise; *R*^*2*^ = .08 for Model 1; *△R*^*2*^ = .04 for Model 2

	***b***	SE ***b***	***β***	***t***	***p***
**Model 1**					
*Constant*	2.55	.16		15.66*	<.001
Age	−0.44	.18	−.24	−2.46*	.02
Relationship Status	−0.24	.16	−.13	−1.51	.14
Employment Status	0.13	.16	.08	0.81	.42
Educational Attainment	0.17	.17	.10	1.02	.31
Duration of Caring	−0.00	.00	−.11	−1.17	.24
**Model 2**					
*Constant*	2.88	.21		13.62*	<.001
Age	−0.42	.18	−.23	−2.40*	.02
Relationship Status	−0.24	.16	−.13	−1.50	.14
Employment Status	0.12	.16	.07	0.73	.46
Educational Attainment	0.22	.17	.13	1.35	.18
Duration of Caring	−0.00	.00	−.11	−1.23	.22
Gender	−0.45	.19	−.21	−2.36*	.02

### Exploratory post-hoc analysis

**Fig. 1:**
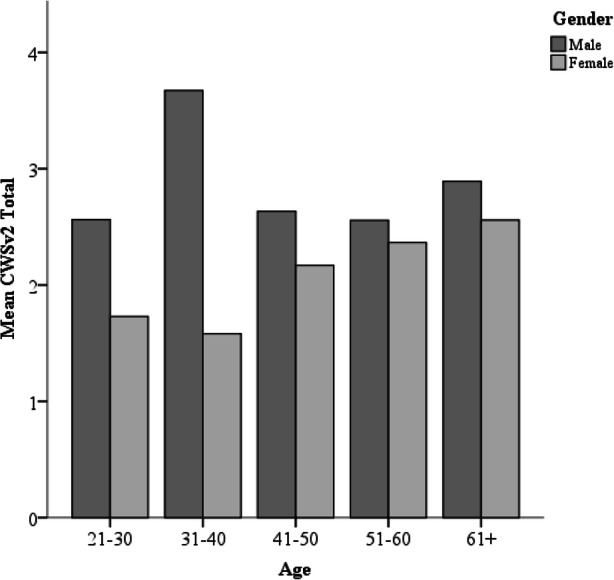
Cluster bar graph of the interaction between carer’s age and gender on carer wellbeing scores. Note: CWSv2 = Carer Wellbeing Scale v2 [30]

The predictors found to be significant in our hierarchical regression were used within a moderation analysis to see if there was an interaction between gender and age on the carer wellbeing. This moderation was run using Hayes (2013) [35] PROCESS macro version 2.16 for SPSS. The total mean score for the CWSv2 was entered as the outcome (Y), with age as the predictor (X), and gender as the moderator (M). For this analysis, we operationalised age as an ordinal variable with 5 levels (1 = aged 21–30; 2 = aged 31–40; 3 = aged 41–50; 4 = aged 51–60; 5 = aged 61+), to enable significant interactions at any of these levels of emerge. The moderation model explained 11.13% of the variance, but the interaction effect was non-significant (*b* = .26; *t* = 1.56, *p* = .12, 95% CI [−.07, .59]). However, when graphing this interaction (see Fig. 1), there is a suggestion that our exploratory analysis represents a Type II error. Figure 1 suggests that while female carers have generally poorer wellbeing compared to males, this difference in wellbeing may be more pronounced for younger carers. Female carers seem to experience an improvement in their wellbeing with age, whereas the wellbeing of male carers worsens after the age of 40. The most vulnerable group therefore appears to be female carers aged 40 and under.

## Discussion

This paper uses secondary analysis of trial data to identify what demographic variables predict the wellbeing of carers of persons with psychosis. Contrary to our hypotheses, being female compared to male, and aged under 50 compared to 50 or over, predicted poorer carer wellbeing. All other variables were non-significant. Our exploratory analysis of the interaction between age and gender was non-significant. However, this may reflect a Type II error. A graph of this interaction suggests that young, female carers report the worst wellbeing.

Our findings are not consistent with previous findings. Studies have found that reduced carer wellbeing was predicted by carers being unemployed (e.g. [24]), single (e.g. [15]), having a lower educational attainment (e.g. [22]), and increased carer age (e.g. [12]) and duration of caring (e.g. [19]). For example, the differences between our results and those found in the aforementioned studies could be attributed to differences in the locality of the participants (i.e. based outside of the UK) (e.g. [15, 24]), and/or the duration of the care recipients’ psychosis (i.e. EIP patients only) (e.g. [12, 22]). Alternatively, our null results with respect to the other predictors could be due to our inclusion of all of the predictors within one regression model. That is, if they were entered into a simple regression then they may have been significant, but when entered into a model with multiple predictors they did not explain sufficient additional, unique variance to be of significance. However, as one of our aims was to test whether predictors had an additive effect, we decided not to explore this possibility. Replicating the study using a more representative sample of carers will enable us to determine whether the null hypothesis should be accepted.

We found that the only significant predictors of carer wellbeing were age and gender. In terms of gender, the majority of our carers were female, which is similar to the gender mix found in other studies of psychosis carers. Like other studies, we found that female carers had poorer levels of wellbeing than male carers (e.g. [13]). With regard to age, the regression slope was in the opposite direction to what we had hypothesised, but this reverse association could be attributed to the validity of our ‘age’ variable. Further inspection of the results from Barrowclough et al. (2014) [36] shows that although older carers were more distressed over the course of their study, having a younger care recipient was also predictive of poor carer wellbeing. In our study, carer age could therefore unintentionally represent the age of their care recipient, with younger carers being more likely to have a younger care recipient. Equally, this discrepancy could be due to differences in the study designs. For example, Barrowclough et al. (2014) [36] found that older carers were the most vulnerable – however, their study only included carers of persons with recent onset psychosis and few carers that represent the ‘oldest old’ age group. However, with the data we currently have, we cannot verify whether either of these explanations is valid or not.

Our exploratory analysis of the interaction between age and gender on carer wellbeing highlights the complexity of this research area. To our knowledge, there are no other studies that have looked at the interaction between predictors. This analytical approach requires a larger sample size, but may yield results with greater clinical applicability. Our moderation analysis was non-significant, but a graphical representation of the data suggests that female carers generally have poorer wellbeing than males, and that this difference is larger for younger carers. The findings from carer research more generally suggest that being young and female could be a proxy measurement for increased carer ‘burden’; as this group tends to provide the most care compared to other age and gender groups [37] - and increased carer burden is associated with worse carer wellbeing (e.g. [23]). However, we must stress that these findings are exploratory and require replication in light of the limitations we have identified below.

### Limitations

Our sample was produced by merging the data sets from two trials; the REACT trial recruited carers of people using EiP services, and the C4C trial recruited only older adult carers. Our population is not representative of the entire carer population as the younger carers of people using longer-term secondary mental health services are not represented. Our findings require replication within the broader carer population before we can conclude whether or not they are generalizable.

Another limitation of conducting secondary analysis of trial data is that our hypotheses and the way in which we conceptualised the predictors was constrained by the way in which the variables were collected in the respective trials. We had to use largely binary categorical predictors collected at a single time point, with uneven between-group sample sizes for the categorical variables. It is therefore possible that we have missed some of the variability within our predictors. For example, we tested whether the carer-care recipient relationship predicted carer wellbeing, but were only able to test parent carers versus non-parent carers; the latter of these groups was heterogeneous, including carers who were the grandparent, sibling, partner, step-parent or friend of the care recipient. These methodological weaknesses can be resolved by the design of a longitudinal study where the hypotheses tested are its primary objective, and the sample size is sufficient to explore both the main and interactive effects of these predictors over time with a greater level of specificity.

### Clinical applications

Carers of persons with psychosis are a vital part of the patient’s care team. The number of carers for people with psychosis is likely to increase in the coming years as both the prevalence of mental health problems [38], and life expectancies [39] increase. The support available to carers in the UK is limited [40], and we therefore need to consider which carers may be most vulnerable, and what kind of support may be most helpful for different carers. Studies like this can help us begin to identify these carers and raise awareness of the different support needs within this population.

The evidence to support a ‘matched care’ approach for carers is not strong enough at present. We must acknowledge that our model only explained 11% of the total variance – this leaves 89% of variance that the demographic variables entered here cannot explain. It is possible that this could be related to one of the limitations of this study, or it could be that demographic variables have limited use in predicting the wellbeing of carers. We advocate for further research that takes into consideration the potential interaction between predictors. It may be more appropriate to take this work forward using a qualitative approach that asks carers what influences their wellbeing. Such an approach could help to identify novel potential predictors.

### Future research

The primary research recommendation is to replicate our findings in light of the limitations identified. If the findings again show that demographic variables only explain a small proportion of the variance, then the limited resources available for carer research should perhaps be redirected elsewhere. Conversely, if the results indicate that demographics have clinical utility, then we recommend researchers turn their attention to the factors that may be maintaining poor wellbeing within that demographic group. Taking this approach will allow clinicians to move away from a ‘one size fits all’ approach, and instead make best use of the limited support available to carers by offering tailored interventions that are more likely to be efficacious. To illustrate, if maladaptive coping strategies [11] were found to be used more by carers of a particular age/gender, then clinicians may recommend these carers engage in a psychoeducation programme aimed at increasing the use of adaptive coping strategies (e.g. Steinhardt & Dolbier, [41]). Secondary to this, and in line with our heuristic approach, there are other demographic factors that could be explored as potential predictors of wellbeing – specifically, recipient characteristics (e.g. patient gender, age, employment status etc.). Depending on the results of these research studies, there may be utility in exploring carer and patient demographics in combination.

## Conclusion

Carers are at an increased risk of developing health problems themselves compared to the general population. Our secondary analysis of trial data sought to identify which carers of persons with psychosis are the most vulnerable in terms of reduced wellbeing. The findings suggest that young carers and female carers experience the poorest wellbeing; with some suggestion that these predictors may interact. This area of research deserves further attention within an appropriately powered study where this is the primary objective. If future research supports our findings, then health professionals can utilise this awareness to inform their clinical decision making and subsequent work with carers.

## Data Availability

The datasets generated and/or analysed during the current study are not publicly available due to their use within the per-protocol analysis for the C4C and REACT trials that has not yet been published; but are available from the corresponding author on reasonable request.

## Abbreviations

C4C: Caring for Caregivers
CWSv2: Carer Wellbeing Scale version 2
EiP: Early Intervention in Psychosis
NHS: National Health Service
NICE: National Institute for Health and Care Excellence
RCT: Randomised controlled trial
REACT: Relative Education and Coping Toolkit
UK: United Kingdom
